# Synthesis and Antimicrobial Studies of New Antibacterial Azo-Compounds Active against *Staphylococcus aureus* and *Listeria monocytogenes*

**DOI:** 10.3390/molecules22081372

**Published:** 2017-08-19

**Authors:** Stefano Piotto, Simona Concilio, Lucia Sessa, Rosita Diana, Gabriel Torrens, Carlos Juan, Ugo Caruso, Pio Iannelli

**Affiliations:** 1 Department of Pharmacy, University of Salerno, Via Giovanni Paolo II 132, 84084 Fisciano (SA), Italy; piotto@unisa.it (S.P.); lucsessa@unisa.it (L.S.); piannelli@unisa.it (P.I.); 2 Department of Industrial Engineering, University of Salerno, Via Giovanni Paolo II 132, 84084 Fisciano (SA), Italy; 3 Department of Chemical Science, University of Napoli “Federico II”, Cupa Nuova Cintia, Napoli 21-80126 , Italy; rosita.diana@libero.it (R.D.); ugo.caruso@unina.it (U.C.); 4 Servicio de Microbiología, Hospital Universitario Son Espases, Instituto de Investigación Sanitaria de Palma (IdISPa), Ctra. Valldemossa 79, 07010 Palma de Mallorca, Spain; gabriel.torrens@ssib.es (G.T.); carlos.juan@ssib.es (C.J.)

**Keywords:** azo-compound, Gram-positive antibacterial, listeria monocytogenes, synthesis

## Abstract

Some novel (phenyl-diazenyl)phenols (**4a**–**m**) were designed and synthesized to be evaluated for their antibacterial activity. Starting from an active previously-synthesized azobenzene chosen as lead compound, we introduced some modifications and optimization of the structure, in order to improve solubility and drug conveyance. Structures of all newly-synthesized compounds were confirmed by ^1^H nuclear magnetic resonance (NMR), mass spectrometry, and UV-Vis spectroscopy. Antibacterial activity of the new compounds was tested with the dilution method against the bacteria strains *Listeria monocytogenes*, *Staphylococcus aureus*, *Escherichia coli*, and *Pseudomonas aeruginosa* PAO1. All the compounds were selectively active against Gram-positive bacteria. In particular, compounds **4d**, **4h**, and **4i** showed the highest activity against *S. aureus* and *Listeria monocytogenes*, reaching remarkable MIC_100_ values of 4 μg/mL and 8 μg/mL. The relationship between antimicrobial activity and compound structure has suggested that the presence of hydroxyl groups seems to be essential for antimicrobial activity of phenolic compounds.

## 1. Introduction

**Figure 1:**
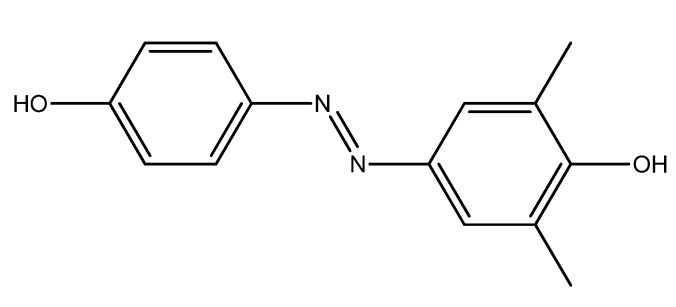
Chemical structure of lead compound **A4** [16].

Substituted azobenzene generally have a color ranging from yellow to red, due to the extensive conjugated system and presence of various electron-withdrawing or electron-donating substituents on the aromatic rings. A key feature of azobenzene and other molecules characterized by a high conjugated pattern is its ability to absorb light with a consequent modification of its structure through the transition from *cis* isomeric form to the *trans* form. This feature is widely-used in light-responsive materials [1,2,3,4,5], liquid crystals [6,7], electronic devices [8,9,10,11], or in NLO chromophores [12,13]. In recent papers, we reported a series of azobenzene-based molecules with antimicrobial properties [14,15,16,17]. For example, studies carried out in our labs showed that **A4** (Figure 1) is one of the most active of the series of compounds synthesized so far, showing antimicrobial activity against *Staphylococcus aureus* (MIC_100_ 20 μg/mL, the Minimum Inhibitory Concentration required to inhibit the growth of 100% of bacteria), against *Candida albicans* (MIC_0_ 17 μg/mL, the Minimum Inhibitory Concentration required to inhibit the growth of 100% of fungi), and *Listeria monocytogenes* (MIC_100_ 25 μg/mL) [16]. 

*Listeria monocytogenes* is one of the most dangerous food pathogens. It shows the ability to survive adverse conditions such as vacuum, low temperature, wide range of pH (4–9), ultraviolet rays and is resistant to conventional pasteurization [18,19,20,21]. Human listeriosis is most often caused by *L. monocytogenes* serogroup 4 [22,23]. The ingestion of products contaminated with this organism may be a potential health threat to high-risk populations, such as the immune-suppressed, pregnant women, and the elderly [24,25]. Azo-compounds that possess antilisterial properties may therefore be expected to become valuable additives as consumer concern with preventing *L. monocytogenes* infections increases.

In an attempt to improve the bioavailability of these molecules, their effectiveness and/or specificity of action, various structural modifications have been proposed of the lead compound **A4**, in order to obtain compounds with better physicochemical properties, while maintaining their antimicrobial activity [26]. In order to evaluate the effect of different substituents on the azobenzene structure, we introduced some new structural changes.

By moving the hydroxyl group from one azobenzene ring to the other and/or adding substituents on both the rings, we obtained compounds with improved antimicrobial activity, with remarkable MIC_100_ value of 10 μg/mL against *S. aureus*, and MIC_0_ of 3 μg/mL against *C. albicans* [26]. Nevertheless, for some new compounds reported here (like **4l**), a tendency to crystallize from aqueous solution was also observed during the period of incubation, and in these cases, it was not possible to reach high concentration of active molecule in the dilution tests. For these molecules, the presence of aromatic rings with hydroxyl groups induces a slow crystallization of the molecule, probably due to the easy packing of the aromatic structures. 

Starting from these observations, we decided to improve the activity of these molecules and to achieve a better understanding of the structure-activity relationship of this class of compounds. The changes include the introduction of alkyl derivatives from four to eight carbon atoms such as butyl, isobutyl, neopentyl, isopentyl, and hexyl moieties, in order to reduce lattice energies in the crystals. The main goal was to prevent the formation of highly ordered crystals that can be an obstacle in reaching the most convenient solubility for pharmaceutical applications.

## 2. Results and Discussion

### 2.1. Chemistry

**Figure 2:**
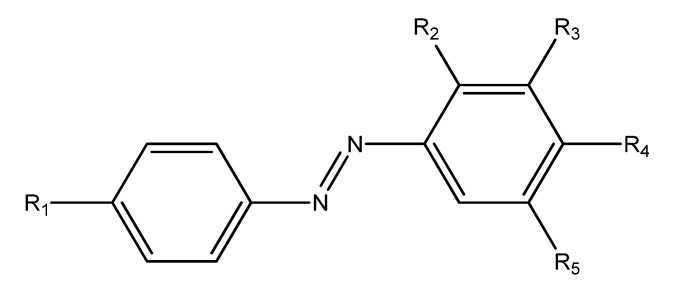
Substituted-(phenyl-diazenyl) phenols derivatives (**4a**–**m**).

The chemical structures of substituted azobenzene derivatives (**4a**–**m**) are reported in Figure 2. 


R_1_R_2_R_3_R_4_R_5_**4a**O(CH_2_)_3_CH_3_HCH_3_OHCH_3_**4b**OCH_2_CH(CH_3_)_2_HCH_3_OHCH_3_**4c**OCH_2_C(CH_3_)_3_HCH_3_OHCH_3_**4d**OCH_2_CH_2_CH(CH_3_)_2_HCH_3_OHCH_3_**4e**OCH_2_CH(CH_2_CH_3_)CH_2_(CH_2_)_2_CH_3_HCH_3_OHCH_3_**4f**HHCH_3_OCH_3_CH_3_**4g**OCH_2_CH(CH_3_)_2_OHHOHH**4h**OCH_2_C(CH_3_)_3_OHHOHH**4i**OCH_2_CH_2_CH(CH_3_)_2_OHHOHH**4j**OCH_2_CH(CH_2_CH_3_)CH_2_(CH_2_)_2_CH_3_OHHOHH**4k**OHOHHOHH**4l**CH_3_OHHOHH**4m**OCH_3_OHHOHH

**Figure 3:**
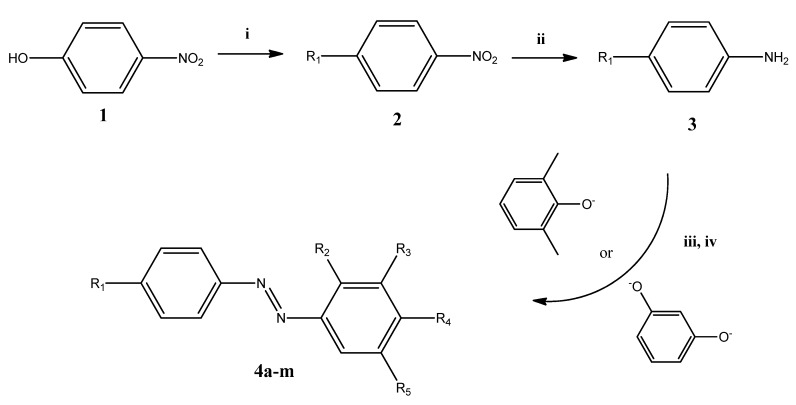
Synthetic route for compounds **4a**–**m**. **i**: Alkylhalide, K_2_CO_3_, DMF reflux, 3 h; **ii**: SnCl_2_, ethanol reflux, 2 h; **iii**: 0–5 °C, NaNO_2_ (aq), HCl 37%; **iv**: 10–15 °C, 1,3-dimethylphenol (for **4a**–**f**) or resorcinol (for **4g**–**m**), NaOH (aq). For R_n_ moieties, see Figure 2.

The synthetic scheme of substituted-azobenzene (**4a**–**m**) derivatives is reported in Figure 3.

### 2.2. Thermal and Optical Properties

In Table 1, thermal and optical properties of the synthesized compounds are reported. Analogues **4b**–**m** showed only a melting peak in the first heating run and they were not able to crystallize from the melt. Only compound **4a**, after the melting peak at 88.7 °C, shows a crystallization phenomenon during the cooling run, at 53.3 °C. When heated in the second run, it showed the same melting peak as the first. Compound **4c** showed three melting peaks in the first heating run at 85.0, 116.7, and 126.0 °C, ascribed to a polymorphism, as confirmed by optical observation under polarized light, but it was not possible to observe any crystallization from the melt.

The spectral region 650–240 nm was investigated by UV-Vis spectrophotometry at a concentration of about 3.0 × 10^−5^ mol L^−1^ of azo-compound in acetonitrile solution (Table 1). The UV-visible spectra for **4a**–**m** are mainly dependent only on the azobenzene unit, which is the same for all compounds. The UV-Vis absorption spectra of all the analogues in solution, showed the typical absorption bands of the electronic transitions of the azobenzene chromophore, with absorption maxima ranging from 360 nm to 447 nm.

### 2.3. Antibacterial Activity

The MIC_100_ of all synthesized compounds was determined by the microbroth dilution method for *Staphylococcus aureus* ATCC 29213, a *Listeria monocytogenes* clinical strain, *Pseudomonas aeruginosa* PAO1, and *Escherichia coli* MG1655 reference strains (Table 2). 

For six of the analyzed compounds, a strong activity against *S. aureus* and *L. monocytogenes* was measured, with MIC_100_ values up to 4 μg/mL and 8 μg/mL, respectively (for **4d**, **4h** and **4i**). To our knowledge, these MIC_100_ values are the lowest ever reached for antimicrobial azobenzene compounds. On the contrary, the activity against gram-bacteria *E. coli* and *P. aeruginosa* was not detectable for any of the synthesized compound, confirming the trend already observed in our previous reports [16,26]. In particular, the high antibacterial activity against *Listeria* is particularly noteworthy, because of the ability of this bacterium to survive in several adverse conditions. Since we do not know the mechanism of action, it is not easy to rationalize the activity of all the analogues **4a**–**m**. Nevertheless, some general considerations can be done, regarding their behavior. The presence of long and disordered aliphatic chains produced the expected effect on the tendency to crystallization for many of the obtained compounds, making them more soluble and available in the incubation medium, compared to analogues **4k**–**m**. For these, it was not possible to achieve high concentration in the dilution tests, due to their easy precipitation from the solution. Another consideration is that by completely removing all hydroxyl groups from the azobenzene moiety, we obtained a complete loss of antibacterial activity (compound **4f**); this indicates that the possibility of making hydrogen bonds is probably fundamental for the interaction of these antibiotics with their target.

## 3. Materials and Methods 

### 3.1. General

All reagents and solvents were purchased from Sigma-Aldrich (Milan, Italy) and used without further purification. Optical observations were performed by using a Jenapol (Zeiss S.p.A., Milano, Italy) microscope fitted with a Linkam THMS 600 hot stage (Linkam, Waterfield, Epsom, Tadworth, UK). Phase transition temperatures and enthalpies were measured using a DSC scanning calorimeter Perkin Elmer Pyris 1 (PerkinElmer, Waltham, MA, USA) at a scanning rate of 10 °C/min, under nitrogen flow. UV absorption spectra of the samples were recorded at 25 °C in acetonitrile solution, on a Perkin Elmer Lambda 19 spectrophotometer. The spectral region 650–240 nm was investigated using a cell path length of 1.0 cm. Azobenzene chromophore concentration of about 3.0 × 10^−5^ mol L^−1^ was used. ^1^H-NMR and ^13^C-NMR spectra were recorded with a Bruker DRX/400 Spectrometer (Bruker, Billerica, MA, USA).

High-resolution mass spectra were acquired on a LTQ-Orbitrap instrument (Thermo-Fisher, Waltham, MA, USA) operating in negative (compounds **4a**–**e**, **4g**–**m**) or positive (compound **4f**) ion mode. Each compound was singularly dissolved in methanol at a concentration of 0.1 mg/mL and injected into the MS ion source. Spectra were acquired in the 150–400 *m*/*z* range.

### 3.2. General Method of the Synthesis of (Phenyl-diazenyl)phenols Derivatives **4a**–**m**

#### 3.2.1. Synthesis of 1-Alkyloxy-4-nitrobenzene (**2a**–**e**, **2g**–**j**)

A suspension of 0.0216 mol of *p*-nitrophenol in 25 mL of DMF was prepared. Then, 6.0 g of K_2_CO_3_ and 0.0264 mol of alkyliodide or bromide were added, and the solution refluxed for 3 h. The solution was then filtered in water and the crude precipitate was recovered and dried under vacuum. Yield ranged between 60 and 90%.

*1-Butoxy-4-nitrobenzene* (**2a**). ^1^H-NMR CDCl_3_: (δ, ppm) = 8.15 (d, 2H), 7.25 (d, 2H), 4.06 (t, 2H), 1.76 (m, 2H), 1.47 (m, 2H), 0.99 (t, 3H). Yield 85%.

*1-(Isobutoxy)-4-nitrobenzene* (**2b**, **2g**). ^1^H-NMR (DMSO-*d*_6_): (δ, ppm) = 8.19 (d, 2H), 7.15 (d, 2H), 3.90 (d, 2H), 2.01 (m, 1H), 0.91 (d, 6H). Yield 80%.

*1-(Neopentyloxy)-4-nitrobenzene* (**2c**, **2h**). ^1^H-NMR (DMSO-*d*_6_): (δ, ppm) = 8.19 (d, 2H), 7.15 (d, 2H), 3.90 (s, 2H), 1.05 (s, 9H). Yield 60%.

*1-(Isopentyloxy)-4-nitrobenzene* (**2d**, **2i**). ^1^H-NMR (DMSO-*d*_6_): (δ, ppm) = 8.16 (d, 2H), 7.10 (d, 2H), 4.10 (t, 2H), 1.78 (m, 3H), 0.91 (d, 6H). Yield 60%.

*1-((2-Ethylhexyl)oxy)-4-nitrobenzene* (**2e**, **2j**). ^1^H-NMR (DMSO-*d*_6_): (δ, ppm) = 8.18 (d, 2H), 7.15 (d, 2H), 4.01 (d, 1H), 3.95 (d, 1H), 1.71 (m, 1H), 1.38 (m, 4H), 1.32 (m, 4H), 0.88 (m, 6H). Yield 65%.

#### 3.2.2. Synthesis of 4-Alkyloxyaniline (**3a**–**e**, **3g**–**j**)

The appropriate 1-alkyloxy-4-nitrobenzene (0.0270 mol) was dissolved in 70 mL of ethanol. Tin chloride (0.119 mol) was dissolved in 30 mL of ethanol and was added dropwise. The reaction was set to take place for two hours at 80 °C. The crude product was extracted twice in a mixture of ethyl acetate/water (1:1), in which 60 g of potassium carbonate was dissolved. The organic solvent was evaporated at reduced pressure and the product was obtained as a yellow oil. Yield ranged between 65 and 85%.

*4-Butoxyaniline* (**3a**). ^1^H-NMR (DMSO-*d_6_*): (δ, ppm) = 6.64 (d, 2H), 6.52 (d, 2H), 4.56 (s, 2H), 3.58 (t, 2H), 1.75(m, 2H), 1.39 (m, 2H), 0.99 (t, 3H). Yield 65%.

*4-Isobutoxyaniline* (**3b**, **3g**). ^1^H-NMR (DMSO-*d*_6_): (δ, ppm) = 6.64 (d, 2H), 6.50 (d, 2H), 4.56 (s, 2H), 3.46 (d, 2H), 2.22 (m, 1 H), 0.96 (s, 6H). Yield 65%.

*4-Neopentyloxyaniline* (**3c**, **3h**). ^1^H-NMR (DMSO-*d*_6_): (δ, ppm) = 6.74 (d, 2H), 6.66 (d, 2H), 4.03 (s, 2H), 3.39 (s, 2H), 1.55 (s, 9H). Yield 85%.

*4-Isopentyloxyaniline* (**3d**, **3i**). ^1^H-NMR (DMSO-*d*_6_): (δ, ppm) = 8.16 (d, 2H), 7.10 (d, 2H), 4.10 (t, 2H), 3.42 (s, 2H), 1.74 (m, 1H), 1.63 (m, 2H), 0.91 (d, 6H). Yield 83%.

*4-((2-Ethylhexyl)oxy)aniline* (**3e**, **3j**). ^1^H-NMR (DMSO-*d*_6_): (δ, ppm) = 6.74 (d, 2H), 6.66 (d, 2H), 4.03 (d, 2H), 3.40 (d, 2H), 1.98 (m, 1H), 1.55 (m, 2H), 1.25 (m, 6H), 0.90 (m, 6H). Yield 85%.

#### 3.2.3. Synthesis of Azobenzene Derivatives **4a**–**m**

Azo compounds **4a**–**m** were synthesized according to the classic scheme of diazo coupling reactions, as reported in ref. [16], and illustrated in Figure 3. The general procedure was the following: 0.0052 mol of the selected alkyloxyaniline (**3a**–**m**) were suspended in a solution containing 4.0 mL of water and 1.1 mL of HCl 37% (*w*/*w*). The solution was cooled at 0–5 °C in a water/ice bath. A solution of 0.42 g of sodium nitrite (0.006 mol) dissolved in 1.2 mL of water was added drop-wise, obtaining a suspension of the diazonium salt (solution A). Separately, a solution containing 1.72 g of NaOH (0.0420 mol) in 16 mL of water with 0.0052 mol of the proper phenol (depending on **4a**–**m**) was prepared (solution B). Solution A was added drop-wise to solution B, under stirring at 12 °C, adjusting the pH at 9–10, with addition of NaOH, if necessary. The system was left reacting for 30 min. Then, 1.2 mL of acetic acid were added, in order to reach a pH value of 5–6, under stirring. A reddish precipitate of the azo compound formed. The crude precipitate was filtered and dried under vacuum. Yields ranged between 45 and 90%.

*4-((4-Butoxyphenyl)diazenyl)-2,6-dimethylphenol* **4a**. 4-Butoxyaniline (**3a**) and 2-6-dimethylphenol were used as starting reagents. The crude product was crystallized from water/ethanol (10:1) to give pure **4a** as gold yellow crystals. Final yield 85%. ^1^H-NMR (DMSO-*d*_6_): (δ, ppm) = 9.01 (broad, 1H), 7.76 (d, 2H), 7.49 (s, 2H), 7.07 (d, 2H), 4.06 (t, 2H), 2.25 (s, 6H), 1.76 (m, 2H), 1.47 (m, 2H), 0.99 (t, 3H). ^13^C-NMR (DMSO-*d*_6_): (δ, ppm) = 163.3; 161.2; 148.5; 146.3; 127.7; 122.7; 116.2; 70.6; 31.3; 20.7; 17.4, 14.7. HR-MS (*m*/*z*): 298.17 [M − H]^−^.

*4-((4-Isobutoxyphenyl)diazenyl)-2,6-dimethylphenol* **4b**. 4-Isobutoxyaniline (**3b**) and 2-6-dimethylphenol were used as starting reagents. The crude product was crystallized from water/ethanol (10:1) to give pure **4b** as yellow crystals. Final yield 90%. ^1^H-NMR (DMSO-*d*_6_): (δ, ppm) = 7.76 (d, 2H), 7.49 (s, 2H), 7.07 (d, 2H), 4.06 (d, 2H), 3.85 (s, 1H), 2.25 (s, 6H), 2.04 (m, 1H), 1.03 (d, 6H). ^13^C-NMR (DMSO-*d*_6_): (δ, ppm) = 163.4; 161.0; 148.6; 146.1; 127.6; 124.6; 124.3; 116.5; 75.0; 27.5; 20.3; 17.2. HR-MS (*m*/z): 298.17 [M − H]^−^.

*2,6-Dimethyl-4-((4-(neopentyloxy)phenyl)diazenyl)phenol* **4c**. 4-Neopentyloxyaniline (**3c**) and 2-6-dimethylphenol were used as starting reagents. The crude product was crystallized from water/ethanol (10:1) to give pure **4c** as dark yellow crystals. Final yield 40%. ^1^H-NMR (acetone-*d*_6_): (δ, ppm) = 7.80 (d, 2H), 7.53 (s, 2H), 7.05 (d, 2H), 4.06 (s, 2H), 2.29 (s, 6H), 1.03 (s, 9H). ^13^C-NMR (DMSO-*d*_6_): (δ, ppm) = 163.6; 161.0; 148.9; 146.0; 127.5; 124.5; 124.0; 117.0; 79.5; 32.6; 26.6; 17.1. HR-MS (*m*/*z*): 312.18 [M − H]^−^.

*4-((4-(Isopentyloxy)phenyl)diazenyl)-2,6-dimethylphenol* **4d**. 4-Isopentyloxyaniline (**3d**) and 2-6-dimethylphenol were used as starting reagents. The crude product was crystallized from water/ethanol (10:1) to give pure **4d** as dark yellow crystals. Final yield 85%. ^1^H-NMR (acetone-*d*_6_): (δ, ppm) = 7.83 (d, 2H), 7.56 (s, 2H), 7.05 (d, 2H), 4.14 (t, 2H), 2.32 (s, 6H), 1.87 (m, 1H), 1.72 (m, 2H), 0.98 (d, 6H). ^13^C-NMR (DMSO-*d*_6_): (δ, ppm) = 163.1; 161.0; 148.3; 146.1; 127.6; 124.6; 115.9; 70.8; 38.0; 28.1; 23.3; 17.2. HR-MS (*m*/*z*): 311.18 [M − H]^−^.

*4-((4-((2-Ethylhexyl)oxy)phenyl)diazenyl)-2,6-dimethylphenol* **4e**. 4-((2-Ethylhexyl)oxy)aniline (**3e**) and 2-6-dimethylphenol were used as starting reagents. The crude product was crystallized from water/ethanol (10:1) to give pure **4e** as orange crystals. Final yield 60%. ^1^H-NMR (acetone-*d*_6_): (δ, ppm) = 7.83 (d, 2H), 7.55 (s, 2H), 7.08 (d, 2H), 4.10 (t, 2H), 2.32 (s, 6H), 1.75 (m, 1H), 1.52 (m, 2H), 1.45 (m, 6H), 0.93(m, 6H). ^13^C-NMR (DMSO-*d*_6_): (δ, ppm) = 163.4; 161.0; 148.6; 146.1; 127.6; 124.5; 124.2; 116.4; 73.4; 39.7; 31.8; 30.5; 24.9; 24.0; 17.2; 14.6; 12.2. HR-MS (*m*/*z*): 354.23 [M − H]^−^.

*1-(4-Methoxy-3,5-dimethylphenyl)-2-phenyldiazene* **4f**. The procedure for obtaining **4f** was a simple methylation of compound **3b**, described in ref [26]. Methyl iodide (0.55 mL) was added to a solution containing 1.00 g of **3b** and 1.22 g of potassium carbonate in 15.0 mL of DMF. The reaction was conducted under reflux for 5 h. The final mixture was then poured in 100 mL of cold distilled water. The organic layer was then extracted in chloroform, dried with sodium sulfate, and the solvent was removed under reduced pressure. Final yield 57%. ^1^H-NMR (DMSO-*d*_6_): (δ, ppm) = 7.95 (s, 2H), 7.85 (s, 2H), 7.62 (m, 3H), 3.74 (s, 3H), 2.33 (s, 6H). ^13^C-NMR (DMSO-*d*_6_): (δ, ppm) = 162.6; 153.1; 148.3; 130.7; 130.0; 129.7; 125.8; 123.0; 61.2; 17.1. HR-MS (*m*/*z*): 241.13 [M + H]^+^.

*4-((4-Isobutoxyphenyl)diazenyl)benzene-1,3-diol* **4g**. 4-Isobutoxyaniline (**3g**) and resorcinol were used as starting reagents. The crude product was purified by column chromatography (ethyl acetate/hexane 3:7) to give pure **4g** as red crystals. Final yield 40%. ^1^H-NMR (Acetone-*d*_6_): (δ, ppm) = 7.85 (d, 2H), 7.71 (d, 1H), 7.10 (d, 2H), 6.59 (d, 2H), 6.40 (s, 1H), 3.88 (d, 2H), 1.05 (d, 6H). ^13^C-NMR (DMSO-*d*_6_): (δ, ppm) = 164.5; 163.4; 161.2; 148.6; 134.6; 125.1; 124.2; 116.4; 110.6; 103.7; 75.0; 27.5; 20.3. HR-MS (*m*/*z*): 286.13 [M − H]^−^.

*4-((4-(Neopentyloxy)phenyl)diazenyl)benzene-1,3-diol* **4h**. 4-Neopentyloxyaniline (**3h**) and resorcinol were used as starting reagents. The crude product was purified by column chromatography (ethyl acetate/hexane 3:7) to give pure **4h** as brilliant red crystals. Final yield 40%. ^1^H-NMR (Acetone-*d*_6_): (δ, ppm) = 7.83 (d, 2H), 7.71 (d, 1H), 7.11 (d, 2H), 6.59 (d, 1H), 6.40 (s, 1H), 3.76 (s, 2H), 1.06 (s, 9H). ^13^C-NMR (DMSO-*d*_6_): (δ, ppm) = 164.5; 163.6; 161.2; 148.9; 134.7; 125.1; 124.0; 117.1; 110.6; 103.5; 79.3; 32.4; 26.4. HR-MS (*m*/*z*): 300.15 [M − H]^−^.

*4-((4-(Isopentyloxy)phenyl)diazenyl)benzene-1,3-diol* **4i**. 4-Isopentyloxyaniline (**3i**) and resorcinol were used as starting reagents. The crude product was purified by column chromatography (ethyl acetate/hexane 3:7) to give pure **4i** as dark red crystals. Final yield 45%. ^1^H-NMR (Acetone-*d*_6_): (δ, ppm) = 7.84 (d, 2H), 7.71 (d, 1H), 7.10 (d, 2H), 6.60 (d, 1H), 6.40 (s, 1H), 4.15 (t, 2H), 1.81(m, 1H), 1.71 (m, 2H), 0.98 (d, 6H). ^13^C-NMR (DMSO-*d*_6_): (δ, ppm) = 164.2; 162.7; 160.8; 147.9; 134.3; 124.7; 124.1; 115.5; 110.3; 103.4; 70.4; 37.6; 27.8; 22.9. HR-MS (*m*/*z*): 300.15 [M − H]^−^.

*4-((4-((2-Ethylhexyl)oxy)phenyl)diazenyl)benzene-1,3-diol* **4j**. 4-((2-Ethylhexyl)oxy)aniline (**3j**) and resorcinol were used as starting reagents. The crude product was purified by column chromatography (ethyl acetate/hexane 3:7) to give pure **4j** as red crystals. Final yield 35%. ^1^H-NMR (Acetone-*d*_6_): (δ, ppm) = 7.80 (d, 2H), 7.67 (d, 1H), 7.08 (d, 2H), 6.56 (d, 1H), 6.36 (s, 1H), 3.98 (d, 2H), 2,01(m, 1H), 1.74 (m, 2H), 1.48(m, 2H), 1.32 (m, 4H), 0,90 (m, 6H). ^13^C-NMR (DMSO-*d*_6_): (δ, ppm) = 163.8; 162.8; 160.5; 147.9; 133.9; 124.3; 123.5; 115.9; 110.1; 103.2; 72.6; 39.1; 31.2; 29.9; 24.3; 23.6; 14.1; 11.6. HR-MS (*m*/*z*): 342.19 [M − H]^−^.

*4-((4-Hydroxyphenyl)diazenyl)benzene-1,3-diol* **4k**. 4-Aminophenol and resorcinol were used as starting reagents. The crude product was crystallized from water/methanol (10:1) to give pure **4k** as red crystals. Final yield 73%. ^1^H-NMR (DMSO-*d*_6_): (δ, ppm) = 10.37, 10.18, 7.74 (d, 2H), 7.62 (d, 1H), 6.91 (d, 2H), 6.50–6.46 (dd, 1H), 6.32 (s, 1H). ^13^C-NMR (DMSO-*d*_6_): (δ, ppm) = 164.1; 160.9; 160.6; 146.8; 134.1; 124.7; 124.4; 116.9; 110.1; 103.1. HR-MS (*m*/*z*): 230.07 [M − H]^−^.

*4-(p-Tolyldiazenyl)benzene-1,3-diol* **4l**. *p*-Toluidine and resorcinol were used as starting reagents. The crude product was crystallized from boiling n-octane and then from water/methanol (10:1) to give pure **4l** as red crystals. Final yield 81%. ^1^H-NMR (DMSO-*d*_6_): (δ, ppm) = 7.63 (d, 2H); 7.50 (d, 2H); 7.44 (d, 1H); 7.34 (d, 2H); 7.27 (d, 2H); 2.33 (d, 3H). ^13^C-NMR (DMSO-*d*_6_): (δ, ppm) = 163.7; 160.4; 149.2; 140.7; 134.1; 129.7; 124.4; 122.7; 110.1; 103.1; 21.3. HR-MS (*m*/*z*): 228.09 [M − H]^−^.

*4-((4-Methoxyphenyl)diazenyl)benzene-1,3-diol* **4m**. 4-Methoxyaniline and resorcinol were used as starting reagents. The crude product was crystallized from boiling n-octane and then from water/methanol (10:1) to give pure **4m** as red crystals. Final yield 45%. ^1^H-NMR (DMSO-*d*_6_): (δ, ppm) = 10.39 (s, 1H); 7.83 (d, 2H); 7.62 (d, 2H); 7.08 (d, 2H); 7.47 (d, 1H); 6.34 (s, 1H); 3.83 (s, 3H). ^13^C-NMR (DMSO-*d*_6_): (δ, ppm) = 163.7; 162.1; 160.4; 147.2; 133.9; 124.4; 114.8; 110.2; 103.2; 56.2. HR-MS (*m*/*z*): 244.08 [M − H]^−^.

### 3.3. Antimicrobial Tests

Bacterial strains and antimicrobial compounds susceptibility determinations.

Four bacterial species were used as models for susceptibility testing; two Gram-negative: *Pseudomonas aeruginosa* PAO1 and *Escherichia coli* MG1655 reference strains; and two Gram-positive: *Staphylococcus aureus* ATCC 29213 strain and a *Listeria monocytogenes* clinical strain isolated in Hospital Son Espases, Palma de Mallorca, Spain. These strains were cultured on blood agar plates, at 37 °C the day before susceptibility determination, which was assessed through the Minimum Inhibitory Concentration (MIC). The MICs of the different antimicrobial peptides were determined by the Müller Hinton Broth (MHB) microdilution method, according to the Clinical and Laboratory Standards Institute (CLSI) guidelines [27]. Briefly, a stock solution of each compound was prepared, following their particular solubility patterns (DMSO, MilliQ water, etc.). This stock solution was then diluted at least 1:50 in MHB to the appropriate concentration of antimicrobial. The MHB containing the appropriate concentrations of antimicrobial compounds was poured into 96-wells plates (100 μL per well), making 1:2 dilutions with fresh MHB on each column of the plate (concentrations ranging from 128 μg/mL to 0.0625 μg/mL). To inoculate each well, 0.5 McFarland bacterial suspensions were prepared in saline solution. Afterwards, 1:100 dilutions in MHB were obtained from those to yield an approx. concentration of 1 × 10^6^ CFU/mL. Finally, 100 μL of these suspensions were used to inoculate the corresponding wells, mixing them with the previously-added MHB containing the antimicrobial compound. Positive (MHB with bacteria but without antimicrobial compound) and negative (MHB alone) controls were routinely prepared in several wells of the plates. Once inoculated, the plates were incubated overnight at 37 °C, and the lowest antimicrobial concentration well in which the bacteria did not grow was considered the MIC. The experiments were always performed in duplicate, in at least two independent occasions, and median MIC values were considered. 

## 4. Conclusions

Some novel derivatives of (phenyl-diazenyl)phenol (**4a**–**m**) were designed, synthesized, and biologically evaluated as antibacterial agents. Many of the synthesized compounds exhibited a significant Gram-positive specific antibacterial activity, in particular against *S. aureus* and *L. monocytogenes*, and they were inactive against Gram-negative bacteria. Three of the synthesized analogues show the best antibacterial activity ever measured for azobenzene compounds. 4-((4-(isopentyloxy)phenyl)diazenyl)-2,6-dimethylphenol (**4d**), 4-((4-(neopentyloxy)phenyl)diazenyl)benzene-1,3-diol (**4h**), and 4-((4-(isopentyloxy)phenyl)diazenyl)benzene-1,3-diol (**4i**) were able to inhibit the growth of 100% of *S. aureus* and *L. monocytogenes* at concentrations three times lower than the lead compound.

The most active antibacterial compounds contain hydroxyl groups, together with long and branched aliphatic chains in their structure. A complete loss of activity was registered for derivative **4f**, which lacks in hydroxyl groups. These observations suggest that the possibility of making hydrogen bonds is probably fundamental for the interaction of these antibiotic molecules with their target receptor.

Thanks to the introduction of particularly bulky and flexible side chains, the molecules presented here could be easily incorporated in DPC micelles, to further increase the drug release. Finally, we could observe that the complete removal of all hydroxyl groups from the azobenzene moiety led to the complete loss of antibacterial activity (compound **4f**).

## Figures and Tables

**Table:** 

	**R_1_**	**R_2_**	**R_3_**	**R_4_**	**R_5_**
**4a**	O(CH_2_)_3_CH_3_	H	CH_3_	OH	CH_3_
**4b**	OCH_2_CH(CH_3_)_2_	H	CH_3_	OH	CH_3_
**4c**	OCH_2_C(CH_3_)_3_	H	CH_3_	OH	CH_3_
**4d**	OCH_2_CH_2_CH(CH_3_)_2_	H	CH_3_	OH	CH_3_
**4e**	OCH_2_CH(CH_2_CH_3_)CH_2_(CH_2_)_2_CH_3_	H	CH_3_	OH	CH_3_
**4f**	H	H	CH_3_	OCH_3_	CH_3_
**4g**	OCH_2_CH(CH_3_)_2_	OH	H	OH	H
**4h**	OCH_2_C(CH_3_)_3_	OH	H	OH	H
**4i**	OCH_2_CH_2_CH(CH_3_)_2_	OH	H	OH	H
**4j**	OCH_2_CH(CH_2_CH_3_)CH_2_(CH_2_)_2_CH_3_	OH	H	OH	H
**4k**	OH	OH	H	OH	H
**4l**	CH_3_	OH	H	OH	H
**4m**	OCH_3_	OH	H	OH	H

**Table 1:** Thermal and optical properties of compounds **4a**–**m**.

Molecule	Thermal Characterization				Optical Characterization	
	T_m_ (°C)	ΔH_m_ (J/g)	T_c_ (°C)	ΔH_c_ (J/g)	λ_max_ (nm)	ε_max_ (L mol^−1^ cm^−1^)
**4a**	88.7	76.5	53.35	66.17	360	26,000
**4b**	126.5	74.3	-	-	360	25,000
**4c**	85.00	52.6	-	-	360	26,100
	116.7	19.5				
	126.0	10.8				
**4d**	74.2	88.4	-	-	361	38,000
**4e**	59.2	56.1	-	-	361	33,100
4f *	-	-	-	-		
**4g**	156.3	54.2	-	-	384	3300
**4h**	179.3	80.2	-	-	384	29,500
**4i**	125.3	73.6	-	-	384	35,700
**4j**	82.6	47.2	-	-	384	3000
**4k**	235	75.4	-	-	382	8300
**4l**	238	91.5	-	-	447	7800
**4m**	160	24.8	-	-	344	4600

**T**_m_ = melting temperature, from DSC analysis, 10 °C/min, nitrogen flow; T_c_ = crystallization temperature, from DSC cooling run; Instrument error ±0.5 °C. ΔH_m_/ΔH_c_ = melting/crystallization enthalpy, evaluated by integration of the peak. Experimental error ±5%. λ_max_ = wavelength at the principal absorption maximum, ε_max_ = molar extinction coefficient at absorption maximum. * Compound **4f** is an oil at room temperature.

**Table 2:** Antimicrobial activity of **4a**–**m** analogues, expressed as MIC_100_ (μg/mL).

MIC_100_ (μg/mL) after 24 h				
	*Staphylococcus aureus*	*Listeria monocytogenes*	*Escherichia coli*	*Pseudomonas aeruginosa*
**4a**	32	>128	>128	>128
**4b**	128	128	>128	>128
**4c**	>128	>128	>128	>128
**4d**	4	8	>128	>128
**4e**	12	48	>128	>128
**4f**	>128	>128	>128	>128
**4g**	>128	96	>128	>128
**4h**	4	8	>128	>128
**4i**	4	8	>128	>128
**4j**	16	16	>128	>128
**4k**	>128	>128	>128	>128
**4l**	>32 *	>32 *	>32 *	>32 *
**4m**	16	16	>128	>128

**M**IC_100_: Minimum Inhibitory Concentration required inhibiting the growth of 100% of organisms after 24 h. The values are the geometric mean of at least three determinations. * Dissolution problems: For these compounds, the initial solution was prepared to achieve 128 μg/mL, but in the end, a maximum MIC of 32 μg/mL was reached, before observing a crystallization of the compound.
